# Ankle proprioception during lateral cutting and stroke performance in adolescent table tennis players

**DOI:** 10.3389/fbioe.2026.1865238

**Published:** 2026-06-25

**Authors:** Ziwei Cao, Mengde Lyu, Shuhui Wang, Jia Han

**Affiliations:** 1 China Table Tennis College, Shanghai University of Sport, Shanghai, China; 2 School of Biomedical Science and Health, Royal Melbourne Institute of Technology University, Melbourne, VIC, Australia; 3 School of Health and Social Care, Shanghai Urban Construction Vocational College, Shanghai, China; 4 School of Exercise and Health, Shanghai University of Sport, Shanghai, China

**Keywords:** adolescent, cutting, performance, proprioception, table tennis

## Abstract

**Objective:**

This study aims to evaluate the association between ankle-cutting proprioception and stroke performance among adolescent elite table tennis players.

**Methods:**

A total of 66 adolescent table tennis players were recruited. Ankle proprioception during lateral cutting movements was assessed using the Ankle Inversion Discrimination Apparatus-Cutting, while sport-specific performance was evaluated through an 80-ball whole-table random placement test (TS80). Hierarchical regression analysis was performed to examine the independent association between ankle proprioception and TS80 scores, controlling for sex, age, and body mass index (BMI).

**Results:**

The baseline model (sex, age, and BMI) significantly predicted stroke performance (R^2^ = 0.26, adjusted R^2^ = 0.23, *p* = 0.0003). Adding ankle cutting proprioception increased the explained variance by 17% (ΔR^2^ = 0.17, *p* < 0.001), with the full model explaining 43% of the variance (R^2^ = 0.43, adjusted R^2^ = 0.39, *p* < 0.001). In the final model, sex (β = 0.55, *p* < 0.001) and ankle proprioception (β = 0.41, *p* < 0.001) were significant independent predictors, while age and BMI were not. A significant sex × proprioception interaction (*p* = 0.012) indicated that the association was stronger in female athletes.

**Conclusion:**

Ankle proprioception during lateral cutting is independently associated with stroke performance in adolescent table tennis players, suggesting that it may be a potential target for future interventional studies aiming at optimizing sport-specific accuracy.

## Introduction

Proprioception refers to the ability of the human body to perceive its own position, movement state, and loading conditions (Han et al., 2016). It plays a fundamental role in maintaining postural control, coordinating movements, and preventing sports injuries (Lephart et al., 1997; Han et al., 2014; Han et al., 2015a). Previous studies have also shown significant correlations between proprioception and performance in multiple sports (Han et al., 2015a; Hams et al., 2019; Lyu et al., 2024). Furthermore, Han et al. (2015b) reported that proprioceptive acuity is closely associated with the competitive level of elite athletes. These suggest that proprioception contributes to talent identification and sport performance. Among all joints, ankle proprioception is particularly important because the ankle and foot complex is usually the first point of contact with the ground (Han et al., 2015a; Lyu et al., 2024).

Table tennis is characterized by high stroke frequency, short rally duration, and variable ball placements (Le Mansec et al., 2017; Shao et al., 2020). Previous studies have shown that table tennis players must perform rapid starts, sudden stops, and lateral displacements within extremely short time frames (Le Mansec et al., 2017; Shao et al., 2020). Specific footwork patterns adjustments allow athletes to rapidly and precisely adjust their body position, helping them enter the optimal hitting space and maintain correct posture during competition (Le Mansec et al., 2017). Stroke stability, shot quality, and tactical execution are directly affected by this ability (Qian et al., 2016). These movements often require cutting actions of the ankle and foot complex (Kovacs, 2009; Shao et al., 2020). These demands highlight the critical role of ankle proprioception, which enables athletes to perceive joint position, movement direction, and loading conditions of the lower limbs accurately (Shao et al., 2020). This perception ensures that footwork movements are timely and precise (Carius et al., 2022).

Previous studies have indicated that ankle proprioception is task-specific (Han et al., 2021; Han et al., 2022). Different proprioception testing methods may reflect distinct capabilities of the brain in processing positional information (Han et al., 2016). Given that table tennis players’ footwork during stroke execution is a dynamic process, it is essential to employ advanced ankle proprioception testing methods with high ecological validity to measure proprioceptive ability during such dynamic movements (Han et al., 2016). Such evaluation may provide useful information for athlete selection and improvement of sport-specific performance.

The purpose of this study was to evaluate ankle proprioception during lateral cutting in table tennis players and examine its relationship with sport-specific performance. Given that sex, age, and morphological characteristics may influence performance in adolescent athletes, these factors were included as covariates in the analysis. We hypothesized that lateral cutting ankle proprioception would be significantly correlated with stroke performance. Adolescence is a period of rapid physical growth and neuromuscular development, during which sex, age, and anthropometric characteristics can influence motor performance (Lyu et al., 2025). Therefore, these variables were entered as covariates in the hierarchical regression to isolate the unique contribution of ankle proprioception.

## Methods

### Participants

*A priori* power analysis using G*Power 3.1 was conducted for a hierarchical linear regression (with four predictors in model 1 and one additional tested predictor in model 2). Assuming a medium effect size (f^2^ = 0.15), an alpha level of 0.05, and a statistical power of 0.80, the minimum required sample size was calculated to be 55. A total of 66 adolescent table tennis players (age: 16.27 ± 3.56 years; height: 166.64 ± 11.81 cm; body mass: 55.05 ± 12.96 kg; body mass index [BMI]: 19.58 ± 2.71 kg/m^2^) were recruited. All participants were offensive attackers utilizing a shakehand grip. All participants were classified as regional elite or national level 2 athletes, actively participating in provincial-level or higher competitions, and possessing at least 6 years of training experience with a minimum weekly training volume of 20 h. None of the participants reported a history of major lower-limb fractures or surgery.

### Ankle inversion discrimination apparatus-cutting

**FIGURE 1:**
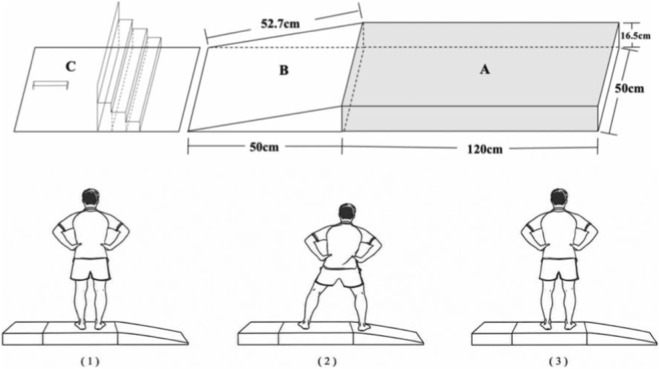
Design and testing procedure of the ankle Inversion discrimination apparatus-Cutting proprioception test. **(A)** Standing platform, **(B)** testing platform, and **(C)** angle adjustment board; lower panel displays the phases of (1) preparation, (2) initiation, and (3) recovery.

Cutting ankle proprioception was measured using the Ankle Inversion Discrimination Apparatus-Cutting (AIDAC), which has been shown to exhibit high reliability in the adolescent table tennis player population (ICC = 0.93) (Cao et al., 2026). AIDAC consists of three main components (Figure 1): the standing platform (A), the testing platform (B), and the angle adjustment board (C). The standing platform is used for preparatory standing before testing and for supporting the non-testing foot during the lateral cutting step. The testing platform is designed to present different ankle inversion angles. During the test, the participant was required to perform a full lateral cutting step and accurately place the testing foot on the testing platform. The angle adjustment board (Figure 1C), used in conjunction with the testing platform, provides four different ankle inversion angles: 10°, 12°, 14°, and 16°.

Before formal testing, a table tennis coach standardizes the lateral cutting step by demonstrating and explaining the procedure to ensure that participants perform the movement correctly during the experiment. The complete cutting step consists of three phases: (1) preparation phase, (2) initiation phase, and (3) recovery phase (Figure 1). During the test, participants must maintain the same posture as during their regular training and retain the same starting and stopping positions, as well as step length, as in the learning phase. After confirming that the participant can perform the standard movement correctly, they proceed to the familiarization phase with AIDAC.

In the familiarization phase, participants stand barefoot on the AIDAC platform with their eyes facing forward. Throughout the entire testing procedure, participants were strictly instructed to maintain a forward gaze and looking down at the testing platform or the angle adjustment board was prohibited. The experimenter and supervising coach closely monitored the participants’ gaze direction during each trial to ensure compliance, thereby eliminating visual feedback and isolating pure proprioceptive pathways. They then complete three familiarization trials to experience the four different ankle inversion angles and learn the correspondence between each angle and the numbers (1 = 10°, 2 = 12°, 3 = 14°, and 4 = 16°). During this phase, participants are informed only of the number–angle correspondence and are not required to provide verbal responses. After completing 12 practice trials, participants proceed to the formal testing phase. In this phase, the experimenter adjusts the inversion angles based on a pre-generated random sequence. Each angle is presented 10 times, for a total of 40 trials. After each stimulus, participants must make an absolute judgment about the perceived inversion angle and verbally report the corresponding number (“1”, “2”, “3”, or “4”). Throughout the formal testing, no feedback regarding the correctness of their judgments is provided. All participants were tested on their dominant limb (Cao et al., 2026). Throughout the test, a coach supervised all trials to ensure that the speed and amplitude of each cutting step remained consistent with those in the initial learning phase. All participants reported no lower-limb injuries within the 6 months prior to testing and had no history of ankle instability or any neurological or musculoskeletal conditions affecting balance.

### Table tennis stroke performance test

**FIGURE 2:**
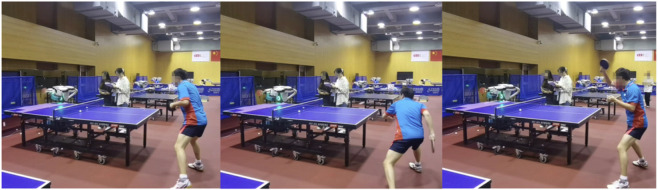
Total score of 80 test procedure.

To ensure consistency and accuracy of ball delivery and closely simulate daily training conditions, this study employed the PongBot, an intelligent table tennis serving robot (Figure 2). The robot allows adjustments of ball placement (zones 1–9), spin type (topspin, backspin, or no spin), ball speed (4–12 m/s), spin rate (0–80 r/s), and frequency (30–85 balls/min). In addition, lateral and longitudinal placement adjustments can be set, enabling the robot to deliver balls with various combinations of spin, speed, and frequency. The maximum ball capacity is 180. Table tennis-specific performance was assessed using the 80-ball whole-table random placement test (total score of 80, TS80). The standard table dimensions were as follows: table surface, 2740 × 1525 mm; height, 760 mm; net width, 1.83 m; and height, 0.1525 m. Each side of the table is bordered by 2 cm-wide white lines, and each half-court is bisected by a 3 mm-wide center line. Following previous studies and simulating match conditions, the opponent’s half-table was divided into three scoring zones (Figure 2): 3-point zone (Zone A): two 30 × 30 cm target areas at the far corners; 2-point zone (Zone B): the remaining one-third of the distal court, excluding Zone A; 1-point zone (Zone C): the rest of the court surface. Shots that failed to cross the net or went out of bounds were scored as 0. A total of 80 balls were delivered, and the cumulative score was calculated as the sum of all strokes according to the scoring zones. To achieve the wide-angle lateral cutting condition, the serving robot was configured as follows: spin type = topspin, ball speed = 6 m/s, spin rate = 2 r/s, frequency = 60 balls/min, one ball per serve, placement zones = 7 and 9, lateral adjustment = 5, and total balls = 80. This specific velocity and dual-zone wide-angle configuration were intentionally selected based on pilot testing to ensure that athletes were required to execute full-amplitude, standardized lateral cutting steps continuously at a match-intensity frequency, avoiding movement distortion (e.g., defensive lunging or sliding) caused by excessive ball speed. During the formal test, participants were required to move according to the randomly placed balls and hit them with maximum effort into the designated scoring zones. Performance was recorded by the examiner. Prior to the formal test, each participant was given one familiarization trial consisting of 30 balls.

### Statistical analysis

Statistical analyses were conducted using R software. Data normality and homogeneity of variance were assessed using the Shapiro–Wilk test and Levene’s test, respectively. Ankle inversion discrimination sensitivity was quantified as the area under the curve (AUC) score (Han et al., 2016). Hierarchical linear regression analysis was performed to examine the predictive effects of the variables on table tennis stroke performance (TS80). In Model 1, sex, age, and BMI were entered to control for demographic and composite morphological factors. In Model 2, ankle proprioception during lateral cutting (AUC) was added to evaluate its independent contribution. To further explore demographic variations, a *post hoc* moderation analysis was conducted by introducing a biological sex-by-ankle proprioception interaction term (sex × proprioception) into the regression framework. For the regression models, unstandardized coefficients (B) with their 95% confidence intervals (95% CI), standardized coefficients (β), and partial coefficients of determination (partial R^2^) as local effect sizes were reported. The change in the coefficient of determination (Delta R^2^) was evaluated using F-change tests. Residual independence and multicollinearity were checked using the Durbin–Watson statistic and variance inflation factors (VIFs), respectively. Statistical significance was set at α= 0.05.

## Results

The hierarchical linear regression analysis revealed that Model 1, containing sex, age, and BMI, significantly predicted table tennis stroke performance (R^2^ = 0.26, adjusted R^2^ = 0.23, F = 7.27, *p* = 0.0003). In Model 2, the addition of the main effect of ankle proprioception during lateral cutting resulted in a significant increase in the explained variance (ΔR^2^ = 0.17, F = 18.04, *p* < 0.001), with the model overall explaining 42.90% of the total variance (R^2^ = 0.43, adjusted R^2^ = 0.39, F = 11.46, *p* < 0.001). Within this model, sex (B = 16.61, 95% CI [10.46, 22.77], β = 0.55, t = 5.40, *p* < 0.001) and ankle cutting proprioception (B = 105.29, 95% CI [55.72, 154.86], β = 0.41, t = 4.25, *p* = 0.0001) were significant independent predictors (Table 1). Collinearity diagnostics confirmed that all VIFs for the main predictors were well within acceptable thresholds (sex = 1.12, age = 1.70, BMI = 1.59, and ankle proprioception = 1.01).

**TABLE 1:** Linear regression model evaluating the interaction of sex and ankle cutting proprioception on table tennis stroke performance.

Predictor	B (95% CI)	SE	Partial R^2^	t	*p*
Sex	−94.04 [-179.60, −8.47]	42.78	0.07	−2.20	0.032
Age	−6.15 [-13.71, 1.41]	3.78	0.04	−1.63	0.109
BMI	0.68 [-0.64, 1.99]	0.66	0.02	1.03	0.306
Ankle proprioception (AUC)	−76.18 [-223.99, 71.63]	73.89	0.02	−1.03	0.307
Sex * proprioception	142.90 [32.66, 253.15]	55.11	0.10	2.59	0.012

Model overall fit: R^2^ = 0.49, adjusted R^2^ = 0.44, F = 11.38, *p* < 0.001. Residual standard error = 11.17. B, unstandardized regression coefficient; SE, standard error; CI, confidence interval; BMI, body mass index; AUC, area under the curve representing dynamic ankle proprioceptive acuity. Sex was coded as 0 = male and 1 = female.

In the final moderation model incorporating the interaction term, the overall framework explained 48.70% of the total variance (R^2^ = 0.49, adjusted R^2^ = 0.44, F = 11.38, *p* < 0.001). The covariates of age and BMI were not significantly associated with stroke performance in the analysis (*p* > 0.05). Crucially, the interaction term between sex and ankle proprioception was statistically significant (B = 142.90, 95% CI [32.66, 253.15], t = 2.59, *p* = 0.012, partial R^2^ = 0.10) (Table 1). Simple slope analysis indicated that the positive relationship between ankle proprioception and stroke performance was more pronounced in female athletes (simple slope = 209.63) than in male athletes (simple slope = 66.72). The final derived regression equation was formulated as follows:

$$\text{TS}80=156.01\;–\;94.04\;*\text{ Sex }–\;76.18\;*\text{ AUC }–\;6.15\;*\text{ Age}+0.68\;*\text{ BMI}+142.90\;*\;\left(\text{Sex }*\text{ AUC}\right).$$

## Discussion

This is the first study to investigate the relationship between ankle cutting proprioception and stroke performance in table tennis players. Hierarchical regression analysis demonstrated that ankle proprioception accounted for an additional 17.9% of the variance in stroke performance beyond developmental and morphological factors, highlighting its independent contribution.

The observed association between ankle proprioception and stroke performance may be linked to the specific biomechanical demands of table tennis. The sport requires frequent, high-intensity, multi-directional changes of direction, where each powerful stroke often originates from lower-limb push-off (He et al., 2021). The ankle, as the terminal joint in contact with the ground, serves as a critical hub for force generation and transmission (Brown et al., 2011; Fuerst et al., 2017; Ding et al., 2024). Superior proprioceptive acuity may theoretically enable athletes to more accurately perceive the position, velocity, and loading of the ankle during high-speed movements (Riemann and Lephart, 2002). From a mechanistic perspective, this enhanced sensorimotor control is hypothesized to optimize footwork precision and provide a foundation for dynamic stability during rapid lateral cutting maneuvers. In table tennis, achieving high stroke accuracy may be modulated by instantaneous micro-postural adjustments at the precise moment of foot–ground contact. Speculatively, accurate somatosensory feedback from the ankle joint could support optimal lower-limb alignment, thereby potentially facilitating a more efficient transfer of ground reaction forces upward through the pelvis and trunk to the upper extremity and racket. Consequently, athletes might be better positioned to maintain a stabilized center of mass and achieve a more consistent racket-ball impact position within a very limited time frame (Iino and Kojima, 2009), suggesting a plausible pathway through which sensorimotor control influences technical precision.

From a practical perspective, the ankle is one of the most frequently injured joints in athletes, and proprioceptive deficits are a well-recognized risk factor for injury. Recurrent ankle sprains not only impair athletic performance but also further reduce proprioceptive function (Brown et al., 2011; Han et al., 2015a). Given the correlational nature of this evidence, these findings should be interpreted as a conceptual framework rather than direct interventional guidelines. Based on the observed strong association, we hypothesize that incorporating targeted dynamic proprioceptive training, such as 2–3 dedicated neuromuscular sessions per week or integrating coordination drills into daily warm-up routines, could serve as an effective pathway for enhancing sport-specific precision (Hübscher et al., 2010; Aman et al., 2015). However, future prospective, longitudinal tracking, and randomized controlled trials (RCTs) are strictly required to test this hypothesis, validate the true causal efficacy of such interventions, and determine optimal training volumes and assessment intervals (e.g., 4–8 weeks) for youth elite athletes (Bahr and Holme, 2003; Read et al., 2018).

Beyond its independent role, biological sex acted as a critical moderator of the relationship between ankle proprioception and stroke performance, as evidenced by the significant interaction effect. Simple slope analysis revealed that the positive association between ankle proprioceptive acuity and stroke accuracy was substantially more pronounced in female athletes than in male athletes. This sex-specific variation may be attributed to distinct neuromuscular control strategies and physical characteristics during adolescence (Lyu et al., 2025). Male athletes generally exhibit greater lower-limb strength and power output, which may enhance their ability to perform rapid directional changes and generate force during stroke execution. Furthermore, differences in motor control strategies and movement efficiency between sexes may also contribute to variations in performance. These findings suggest that stroke performance is jointly influenced by biological characteristics and sensorimotor function. However, given the relatively modest sample size, these sex-specific moderation findings should be interpreted with caution and regarded as exploratory until replicated in larger cohorts.

Several limitations should be acknowledged. The study design is strictly cross-sectional and lacks longitudinal tracking or interventional data, which precludes causal inferences regarding whether enhancements in ankle proprioception directly drive improvements in table tennis stroke performance. Therefore, our conclusions regarding training applications remain speculative and serve purely as foundational hypotheses for future interventional research. The TS80 assesses stroke accuracy under random placement conditions but does not capture ball speed, spin, or tactical decision-making, all of which are components of match performance. Third, participants were tested in a single session without assessment of intra-individual variability or fatigue effects; however, the 80-ball protocol is relatively short (∼5–7 min) and unlikely to induce significant fatigue. All participants wore their own table tennis shoes, which may have introduced variability in proprioceptive feedback; future studies should standardize footwear. The examiner was not blinded to participants’ performance levels, which could introduce bias, though the robot-based TS80 scoring is objective. Finally, the generalizability of these findings to non-elite or younger athletes, or to other racquet sports, remains to be established.

## Conclusion

Ankle proprioception during lateral cutting is independently associated with stroke performance in adolescent table tennis players. Although these cross-sectional findings demonstrate strong predictive value beyond morphological factors, future longitudinal research is needed to confirm whether targeted proprioceptive training directly leads to improvements in stroke accuracy.

## Data Availability

The raw data supporting the conclusions of this article will be made available by the authors, without undue reservation.
